# Echoentomography for Assessing Braconid Parasitization on Soft-Bodied Tephritid Hosts

**DOI:** 10.3390/insects12110980

**Published:** 2021-10-29

**Authors:** Renato Ricciardi, Rossana Izzetti, Marco Romanelli, Davide Caramella, Andrea Lucchi, Giovanni Benelli

**Affiliations:** 1 Department of Agriculture, Food and Environment, University of Pisa, Via del Borghetto 80, 56124 Pisa, Italy; renato_ricciardi@hotmail.it (R.R.); andrea.lucchi@unipi.it (A.L.); 2 Unit of Dentistry and Oral Surgery, Department of Surgical, Medical and Molecular Pathology and Critical Care Medicine, University of Pisa, 56124 Pisa, Italy; ross.izzetti@gmail.com; 3 Unit of Dermatology, Department of Clinical and Experimental Medicine, University of Pisa, 56124 Pisa, Italy; marco.romanelli@unipi.it; 4 Diagnostic and Interventional Radiology, Department of Translational Research and of New Technologies in Medicine and Surgery, University of Pisa, 56124 Pisa, Italy; davide.caramella@unipi.it

**Keywords:** Braconidae, *Ceratitis capitata*, insect imaging, parasitoid mass rearing, *Psyttalia concolor*, Tephritidae, ultra-high frequency ultrasound (UHFUS) technology

## Abstract

**Simple Summary:**

Host parasitization by an endoparasitoid insect can be evaluated relying on three main methods, i.e., dissection under a stereomicroscope, polymerase chain reaction (PCR), or waiting for adult emergence. These approaches show limitations related to the time required for the detection of parasitization and/or the destruction of the sample. In this research, an innovative approach using ultra-high frequency ultrasound (UHFUS) technology was employed to evaluate braconid parasitization of *Ceratitis capitata* larvae. The UHFUS approach was compared with classic stereomicroscopic dissection, showing that both methods provide comparable diagnostic reliability. Our results support the application of echoentomography as a useful tool for fine, fast, and non-invasive evaluation of the ability of parasitoids to parasitize soft-bodied hosts.

**Abstract:**

Entomological approaches currently available for assessing host parasitization require dissection, polymerase chain reaction (PCR), or waiting for adult emergence. The first two methods are relatively fast but destructive, whereas the third one allows the emergence of the parasitoid but it is time consuming. In this framework, new diagnostic imaging tools may contribute to solve the lack of an accurate, rapid, and non-invasive approach to evaluate the parasitization of soft-bodied insects by their endoparasitoids. In this study, ultra-high frequency ultrasound (UHFUS) technology, which is currently used in medical and preclinical fields, was adopted to assess the parasitization of the invasive polyphagous Mediterranean fruit fly, *Ceratitis capitata* (Diptera: Tephritidae), testing 2nd and 3rd instar larvae. Parasitization assays were carried out with the solitary koinobiont endophagous parasitoid *Psyttalia concolor* (Hymenoptera: Braconidae: Opiinae). The efficacy of UHFUS-based echoentomography was compared with the classical method of dissecting the larval host under a stereomicroscope. Our results showed that the UHFUS diagnostic capability was statistically comparable with that of dissection, both on *C. capitata* 2nd and 3rd larvae. Overall, UHFUS-based echoentomography may be further considered as a fast, non-invasive, and effective approach to evaluate the parasitoid’s ability to successfully oviposit in soft-bodied hosts.

## 1. Introduction

Recently, technologies such as radiography, computed tomography (CT) and micro-CT, magnetic resonance imaging (MRI), and ultrasonography, which were previously strictly dedicated to medical and pre-clinical research, have been considered for studying insect morpho-physiology, achieving fascinating results [1,2,3]. Indeed, these studies gave detailed insights about the functional anatomy of the insect’s brain [4,5], muscles [1], exoskeleton, and respiratory system [3].

Assessing the parasitization success of mass-reared hymenopteran parasitoids on their potential hosts is of crucial importance for developing inexpensive and reliable large-scale production of biological control agents (BCA) [6,7]. Current methods to detect parasitization efficacy on a given insect host are time-consuming and/or destructive, mainly based on waiting for the parasitoid emergence, if the parasitoid’s egg is not encapsulated by the host’s immune system, on anatomical dissection of the host under the stereomicroscope [8], and on polymerase chain reaction (PCR) to detect and identify parasitoid DNA [9,10]. In this framework, fast-track non-invasive methods are needed, with special reference to rearing of rare or hard-to-maintain species of potential biocontrol relevance.

The present study aims to provide a novel fast-track approach, allowing real-time detection of successful parasitoid oviposition on a given host, relying on ultra-high frequencies ultrasound (UHFUS) technology. This is a novel implementation of the conventional ultrasound technology, allowing visualization of tiny anatomical details, thanks to advanced UHF transducers, to reach outstanding spatial resolution at frequencies up to 100 MHz [2,11,12,13,14]. As a study model, we focused on the trophic interaction between the endoparasitoid *Psyttalia concolor* (Szépligeti) (Hymenoptera: Braconidae) and its host, the Mediterranean fruit fly (medfly), *Ceratitis capitata* (Wiedemann) (Diptera: Tephritidae), an invasive polyphagous species able to attack over 250 plant species [15]. *Psyttalia concolor* is a synovigenic koinobiont larval-pupal endoparasitoid. This solitary parasitic wasp attacks 2nd–3rd instar larvae of at least fourteen tephritid species [16], including the medfly and the olive fruit fly, *Bactrocera oleae* (Rossi) (Diptera: Tephritidae) [16]. To cope with the above-mentioned challenges, herein a non-invasive UHFUS-based approach allowing real-time detection of the parasitoid’s egg in a living host was proposed, assessing the oviposition success of *P. concolor* in *C. capitata* larvae. The reliability of UHFUS-based echoentomography was compared with that of traditional invasive dissection under a stereomicroscope.

## 2. Materials and Methods

### 2.1. Insect Rearing

*Ceratitis capitata* adults were mass-reared in cylindrical PVC cages (Ø = 35 cm; h = 35 cm) at a density of about 2000 flies per cage. Medflies were fed on a dry diet composed by sucrose and yeast extract (Sigma-Aldrich, Germany) (10:1, w:w); water was provided on a cotton wick through dedicated dispensers. Larvae were reared in plastic bowls (50 × 15 × 2 cm) containing 0.5 kg of an artificial culture medium (i.e., bran, devitalized yeast, wheat germ, and sugar), as detailed by Canale and Benelli [16]. Our medfly rearing is maintained in laboratory conditions at 23 ± 2 °C and relative humidity (RH) of 45 ± 5%.

*Psyttalia concolor* young instars were reared on 3rd instar medfly larvae, according to Canale and Benelli [16]. *Psyttalia concolor* adults were fed ad libitum on a mixture of honey and bee-collected pollen in small cylindrical vial caps (0.5 × 2 cm). Water was provided separately on a cotton wick [17].

### 2.2. Host Parasitization Protocol and UHFUS Observations

**Figure 1:**
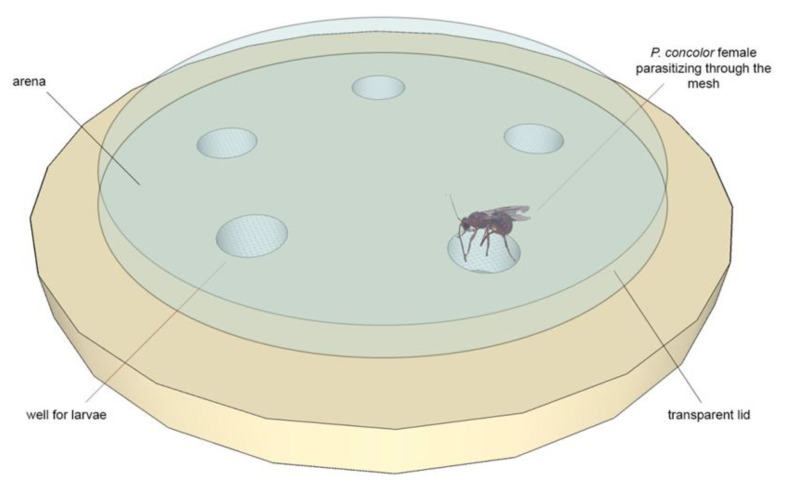
A schematic representation of the arena proposed by Benelli et al. [17] for tephritid parasitization by braconid larval-pupal endoparasitoids; each well was filled with a single larva plus its rearing medium. Then, a parasitoid female was allowed to parasitize the larva through the mesh holes covering the wells, mimicking the fruit’s surface.

Using the multiple-well arena described by Benelli et al. [17], covered with a transparent Petri dish lid (Ø = 55 mm; h = 13 mm) (Figure 1), forty 2nd and 3rd instar *C*. *capitata* larvae were parasitized by *P. concolor* females. Second and third instar medfly larvae were empirically identified according to their size and the number of days since the eggs hatched at a known temperature [16]. Each medfly larva was parasitized individually by a *P*. *concolor* female released in the above-mentioned arena; each larva was considered successfully parasitized when the parasitoid kept the ovipositor in the host for at least 20 s [18]; for this reason, the host’s exposure to the parasitoid was directly monitored by an observer.

For each selected instar, 40 larvae were analyzed through UHFUS in vivo observations using the Vevo^®^ MD (Fujifilm, VisualSonics, Toronto, ON, Canada) 70 MHz transducer, and following the method adopted by Ricciardi et al. [2]. The 70 MHz transducer (UHF 70) is characterized by a 30 µ axial resolution, which makes it suitable for the investigation of structures of extremely small size. Therefore, plastic wells slightly larger than the larvae (8.4 × 2.6 × 5 mm) were used to minimize larvae movements without blocking them. The probe was locked to an adjustable support, 2–3 mm above the study insect included in sterile ultrasound gel (Teleflex Medical, Varedo, Italy); 2D mode scans were carried out. After each analysis, the larvae were gently washed with distilled water and separated into vials to allow parasitoid development. The remaining 40 larvae, representing the control, were routinely dissected in 70% ethanol under the stereomicroscope (Greenough S9e, Leica Microsystems, Wetzlar, Germany) searching for *P*. *concolor* eggs. All trials were conducted from July to September 2020.

### 2.3. Statistical Analysis

The reliability of UHFUS-based echoentomography in detecting a successful parasitoid oviposition (estimated in terms of egg no. detected in each medfly host) was compared with classic dissection using a contingency analysis. JMP 11 (SAS) was used for the analyses.

## 3. Results

**Figure 2:**
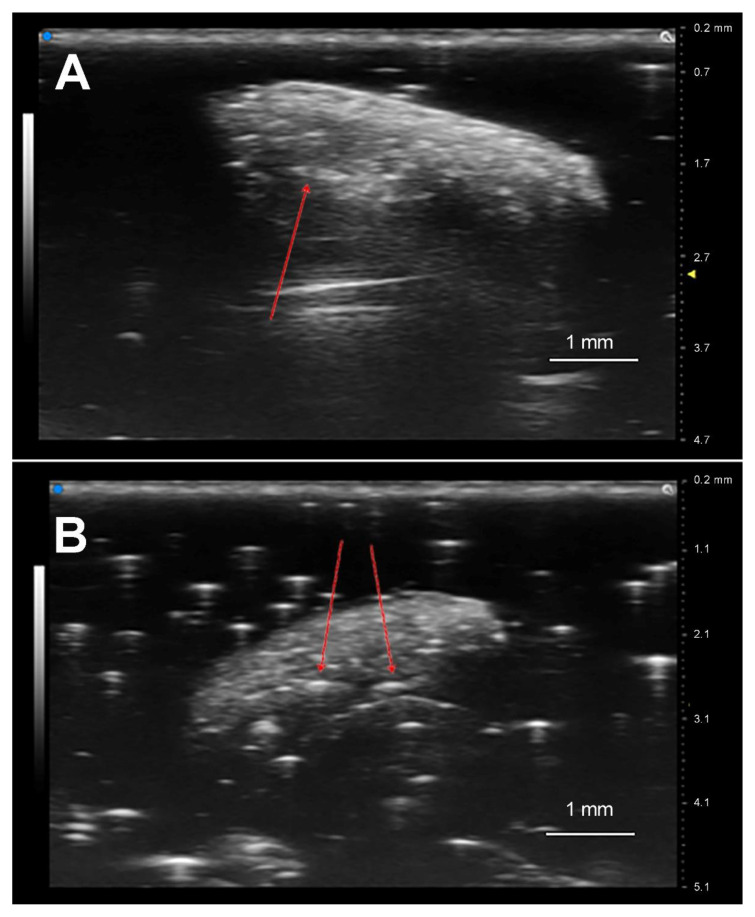
Ultrasound scans, 6 h after parasitization, showing the presence of (**A**) an egg of the braconid parasitoid *Psyttalia concolor* in a 3rd instar of *Ceratitis capitata* and (**B**) two eggs of the same parasitoid in a 2nd instar medfly larva.

Relying on the UHFUS technology, it was possible to observe the presence of one (Figure 2A) or more *P. concolor* eggs (Figure 2B) within the medfly host, thus confirming the parasitization event. The analysis of each sample required a short time; indeed, within 3 to 5 min it was possible to assess the presence or absence of parasitoid eggs (laid 6 h before) in the *C*. *capitata* larval body. The eggs appear slightly curved in shape, with a whitish nucleus immersed in cytosol (black area surrounding the core), all contained by the chorion.

**Figure 3:**
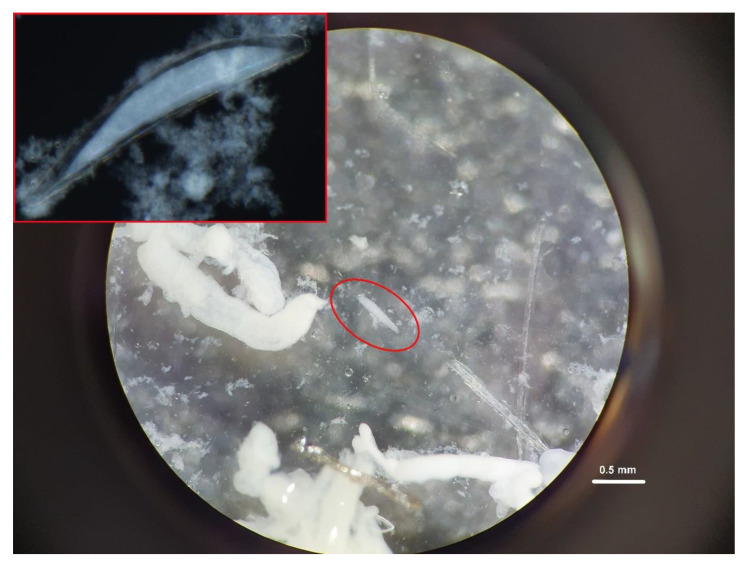
Eggs of *Psyttalia concolor* (marked by red circle) detected through dissection in 70% ethanol of a 2nd instar of *Ceratitis capitata,* 6 h after parasitization. In the left corner of the figure, an enlarged view of the parasitoid egg.

**Figure 4:**
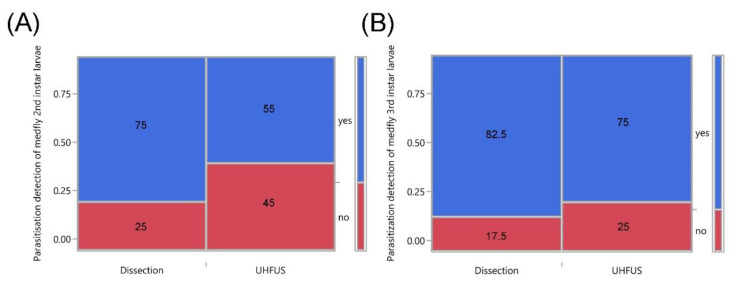
Contingency analysis comparing the diagnostic efficacy of ultrasonography (UHFUS) and stereomicroscopic dissection carried out on (**A**) 2nd and (**B**) 3rd instar larvae of *Ceratitis capitata* parasitized 6 h before by *Psyttalia concolor*. The bar on the right represents the relative abundance of successful parasitization events compared with the total number of tested medfly larvae (blue—yes, indicates the larvae where one or more eggs of the parasitoid have been detected; red—no, indicates the larvae where no eggs of the parasitoid have been detected). The number in each box indicates the percentage of parasitized medfly larvae.

Microscopic dissection of the parasitized medfly larvae confirmed the gross morphology of the braconid parasitoid egg, as observed through UHFUS (Figure 3). Comparing the two techniques to evaluate successful parasitization of second (*χ^2^* = 3.55, *d.f.* = 1, *p* = 0.059) and third (*χ^2^* = 0.675, *d.f.* = 1, *p* = 0.411) instar medfly larvae, no significant difference was noted between the diagnostic accuracy of the two techniques (Figure 4A,B). Moreover, through UHFUS-based echoentomography, it was possible to assess, quite simply, the size of the eggs, corresponding to 0.49 ± 0.02 mm (length, mean ± SD).

## 4. Discussion

Currently, UHFUS technology is mainly used in the medical field, in particular for the evaluation of cutaneous structures, which makes the technique extremely performing in dermatology and oral medicine [19,20]. However, in recent years it has been employed for preclinical studies on model animals such as zebrafish [21] and rodents [22,23]. Of note, high-frequency ultrasound-based approaches can be of interest for studying soft-bodied insects in vivo, leading to a novel research approach recently named as echoentomography [2]. On the other hand, insects characterized by a solid chitinous exoskeleton are poorly suitable for this kind of investigation due to the difference in acoustic impedance that causes the reflection of most waves at the boundary between gel and their exoskeleton, not allowing a clear analysis of the tissues. In the case of soft-bodied insects, UHFUS-based echoentomography can allow the observation of the morpho-physiology of economically important insect species, simultaneously preserving the viability of the individual. For example, a recent study relying on UHFUS-based echoentomography allowed the in vivo analysis of *C. capitata* and *Lobesia botrana* (Denis & Schiffermüller) (Lepidoptera: Tortricidae) larvae, allowing the quantification key anatomical and physiological features, such as the pyloric valve rotating movements and haemolymph flux, just to cite some [2].

In our opinion, UHFUS-based echoentomography may also be used for real-time studies to assess the impact of biotic and abiotic stressors, such as selected insecticides used to control the soft-bodied juvenile stages of a given pest species. Furthermore, investigations of host–parasite interactions could be carried out using this approach. On this last assumption, herein we compared the reliability of echoentomography and stereo-microscopic dissection, to detect the eggs laid by *P. concolor* females in the soft body of *C. capitata* 2nd and 3rd instar. Our results revealed the possibility of assessing the host parasitization in vivo with an accuracy comparable with stereomicroscopic dissection, and a faster processing time for each tested individual.

Currently, three main tools are used to assess the potential successful parasitization of a target insect: (*i*) waiting for the parasitoid emergence from the host [24,25], (*ii*) dissecting the host to assess the presence of host eggs [26,27], or (*iii*) using PCR to detect and identify parasitoid DNA [9,10]. The first approach is inexpensive and not destructive, but slow since parasitization can be confirmed only after several days or weeks. The second approach is equally inexpensive, parasitization can be quickly detected, but the overall analysis is invasive for both the involved individuals. The PCR-based approach is efficient, but destructive and more expensive compared with the above-mentioned ones [28]. Compared with these approaches, UHFUS-based echoentomography is rapid, effective, non-invasive, and allows in vivo analysis. However, the latter is limited to observations on the larval stage of the host, because the study of hard-bodied insects or chitinous structures such as the pupa proved to be impossible, due to the total reflection of the ultrasound wave by the exoskeleton [2]. Moreover, it is also inexpensive when performed on equipment already available in the clinical setting, which can be accessible for non-clinical research at little or no additional costs. Indeed, the introduction of UHFUS for clinical use is very recent, and medical applications and clinical research are not able to saturate the equipment yet, making it freely available for non-clinical research.

It should be noted that the exclusive use of UHFUS for entomological purposes may not be justified if the volume of examinations is limited, due to the high acquisition and maintenance costs of the equipment. As a matter of fact, the cost of dissecting microscopes is much lower when compared with UHFUS. Nevertheless, the distinct advantage provided by UHFUS in terms of reduced invasiveness and the possibility to perform in vivo analysis appears very promising, and we foresee a growing application of the technique in entomology. Therefore, establishing a collaboration between research groups and clinical centers already equipped with UHFUS may help to overcome such a limitation.

## 5. Conclusions and Challenges for Future Research

Overall, relying on UHFUS-based echoentomography for assessing successful parasitization of soft-bodied hosts by hymenopteran endoparasitoids allows rapid, reliable, and non-invasive assessment of the parasitization success during the larval phases of the host. Moreover, the proposed methodology may represent a valuable tool to finely track the development and the morpho-physiology of parasitoid preimaginal stages developing in soft-bodied larval hosts. The opportunity to analyze and monitor insect morpho-physiology in vivo may have an important application value, allowing the non-invasive study of difficult-to-collect or endangered species, as well as the investigation of key lethal and sub-lethal effects due to insecticide exposure.

## Data Availability

Data are contained within the article.
